# Microbial metabolites as modulators of the infant gut microbiome and host-microbial interactions in early life

**DOI:** 10.1080/19490976.2023.2192151

**Published:** 2023-03-21

**Authors:** Henrik M. Roager, Catherine Stanton, Lindsay J. Hall

**Affiliations:** a Department of Nutrition, Exercise and Sports, University of Copenhagen, Frederiksberg, Denmark; b APC Microbiome Ireland, Teagasc Moorepark Food Research Centre, Fermoy, Co. Cork, Ireland; c Gut Microbes & Health, Quadram Institute Biosciences, Norwich, UK; d Intestinal Microbiome, School of Life Sciences, ZIEL – Institute for Food & Health, Technical University of Munich, Freising, Germany; e Norwich Medical School, University of East Anglia, Norwich, UK

**Keywords:** Metabolites, metabolomics, infants, microbiota, health, host, diet

## Abstract

The development of infant gut microbiome is a pivotal process affecting the ecology and function of the microbiome, as well as host health. While the establishment of the infant microbiome has been of interest for decades, the focus on gut microbial metabolism and the resulting small molecules (metabolites) has been rather limited. However, technological and computational advances are now enabling researchers to profile the plethora of metabolites in the infant gut, allowing for improved understanding of how gut microbial-derived metabolites drive microbiome community structuring and host-microbial interactions. Here, we review the current knowledge on development of the infant gut microbiota and metabolism within the first year of life, and discuss how these microbial metabolites are key for enhancing our basic understanding of interactions during the early life developmental window.

## Introduction

A relatively simple microbial community at birth develops into a diverse and complex microbial ecosystem across the early life window^1^, which plays a vital role in postnatal maturation and development of the endocrine, nervous and immune systems^2,3^. The gut microbiome thus has a profound influence on both early and long-term health and development^4,5^. In addition to influencing development and maturation of the host immune system, the gut microbiota has a number of important functions in the host, including breakdown of ingested complex carbohydrates, protection against pathogenic bacteria and metabolic functions including production of vitamins, short chain fatty acids (SCFAs) and bile acid biotransformation^6^. The importance of neonatal bacterial colonization for the growth of a healthy individual is clear and exhibits the power that the microbiome has on its host from an early stage in development.

The gut microbiota composition is initially known to be in a state of flux and consequently stabilizes, so that by age 2.5–4 years, it begins to resemble the composition and characteristics of an adult gut^7,8^. In this early phase, the gut microbiota is more vulnerable to modulation than in the adult state^9^, suggesting that the first 1000 days of life is a critical window of opportunity for optimal bacterial colonization. Disruption to microbial development during this period is believed to have long-lasting consequences, impacting on normal immune and neurocognitive development and has been associated with increased risk of developing diseases such as asthma and metabolic syndrome later in life^10,11^.

The gut microbiota may also be modulated at this critical phase in a beneficial manner. Strategies to optimize early microbial acquisition during this key developmental window of opportunity may help to prevent and treat disease. Interventions to modulate the early microbiome include the use of probiotic and prebiotic interventions or combinations of both, as either synbiotics^12^ or postbiotics^13^. The most promising results for probiotics have been seen in the prevention or treatment of diarrhea in children and in necrotizing enterocolitis in preterm infants^14,15^. Pharmabiotics including microbially produced bacteriocins, bacteriophages and SCFAs have gained interest for potential application for microbiota modulation^16–19^. Furthermore, there has been a shift in recent years to expand microbiome research beyond profiling taxonomic composition using 16S rRNA gene sequencing and to focus on gut microbial functionality and activity using whole metagenome sequencing (WMS) and metabolomics. WMS while more costly than 16S rRNA sequencing provides insight on the functions encoded by the genes present in the microbiome. WMS also facilitates community structure to be examined at a far greater and reliable taxonomic resolution beyond genus level to examine species and subspecies^20^. In addition, metabolomics is a useful complementary approach to measure metabolites in the gut derived from the diet, host or the microbiota (collectively denoted the gut metabolome)^21^. Advances in informatics such as machine learning have also provided superior insight and facilitated meaningful interpretations of functional microbiome research^22,23^. This review briefly discusses the factors influencing the gut microbiota in early life, summarizes current literature available on the diet-dependent development of the infant gut metabolome, and discusses how microbiota-derived metabolites may be key for early life microbiome and host development.

## Factors influencing the gut microbiota in early life

### Non-nutritional factors

Delivery mode exerts a significant influence of gut microbiota composition, exposing the infant to various microbes associated with the maternal gut and vaginal tract^24^. Passage of the infant through the birth canal exposes it to the mother’s vaginal and fecal bacteria thereby contributing to microbial and metabolic diversity in the infant gut^25^. Cesarean section impairs the transfer of certain maternal strains resulting in an infant gut microbiota with increased abundance of potentially pathogenic microbes, and a lack of beneficial taxa such as *Bifidobacterium*^26^. Other factors reported to influence the development of the infant gut microbiome include gestational age, host genetics, feeding regime, geographical location and perinatal antibiotic usage^27,28^. Gestational age is an important factor in gut microbiota development with preterm birth associated with reduced diversity and delayed colonization with *Bifidobacterium* compared with full-term infants^29^. Exposure to antibiotics in early life has been reported to induce an increased abundance of members of the *Enterobacteriaceae* family and decrease in abundance of *Bifidobacterium* ^30,31^. Antibiotic exposure has also been linked with the development of metabolic and inflammatory diseases later in life^32^. In recent years, evidence also supports the existence of a breast milk microbiome, which is a source of beneficial microbes to the infant^27,33,34^. Infants also acquire microbes that colonize their gut from the environment including siblings, pets and the hospital^5,28,35^.

### Breastmilk

Human breast milk can meet all nutritional requirements essential for infant growth, health and development^36^. Breast milk contains essential nutrients such as caseins, whey, fatty acids, and lactose as well as micronutrients including Immunoglobulin A, Immunoglobulin G, Immunoglobulin M, calcium and vitamin A^28,37^. Furthermore, breast milk is a source of an array of bioactive components such as human milk oligosaccharides (HMOs). HMOs consist of > 150 known complex glycans with the most common being 2′-fucosyllactose (2′FL), accounting for 20–30% of all HMOs^37–39^. HMOs are not digested in the upper gastrointestinal (GI) tract and are degraded in the colon by specific HMO-degrading bacteria, mostly *Bifidobacterium*, into metabolites such as acetate and lactate with potential beneficial host health effects^28,38^. The ability of some species of *Bifidobacterium* to efficiently degrade HMOs makes them dominant members of the microbiota of breastfed infants. Host secretor status, based on active or inactive copy of the fucosyltransferase 2 (FUT2) gene, determines a mother’s ability to produce HMOs^40–43^. Non-secretors have been associated with reduced *Bifidobacterium* and *Bacteroides* abundances and lower microbial diversity in their infant’s gut microbiota^44–47^. The absence/decrease of 2′FL in non-secretor women has potential implications for infant short and long term health and development^48–50^.

### Formula milk

Compositional analysis of the infant gut microbiota has shown altered community structures in formula-fed infants compared to breastfed infants^51,52^. Higher abundance of potentially pathogenic bacteria such as *Enterobacteriaceae* and *Clostridium*, as well as antimicrobial resistance genes, is observed in formula-fed infants compared to exclusively breastfed infants^8,51,53,54^. Conversely, breastmilk feeding is associated with low microbial diversity, dominated by HMO-utilizing *Bifidobacterium* species and saccharolytic fermentation compared to formula-feeding^27,55^. Due to formula milk’s higher protein content, formula fed infants may also exhibit a higher degree of proteolytic metabolism in the gut^56,57^. Proteolytic microbiota-derived metabolites have been linked to detrimental cardiovascular and metabolic health effects in adults^58–61^, as discussed in greater details below. Yet, whether changes in colonic fermentation observed in formula fed infants influence the risk of developing diseases later remains unknown. Nonetheless, many infant formulas have in recent years been supplemented with prebiotics in an attempt to mimic health benefits of HMOs in breast milk. Prebiotic supplementation has predominantly consisted of short-chain galacto-oligosaccharides (scGOS), long-chain fructo-oligosaccharides (lcFOS), and HMOs such as 2-fucosyllactose and Lacto-N-neotetraose^62–64^.

### Weaning and beyond

During the transition period to solid foods, typically between 4–6 months of age, changes appear in the richness and diversity of the infant gut microbiota. Milk adapted bacterial species such as *B. longum* subsp. *infantis* typically decrease in this period^65^. Due to the increased consumption of protein and dietary fibers during this period, microbes capable of digesting more complex nutrients increase in abundance such as *Lachnospiraceae*, *Bacteroidaceae* and *Ruminococcaceae*^66–68^. Microbiota maturation occurs continuously even beyond five years of age where the children have acquired a diverse, stable and adult-like configuration, but with a lower community richness compared to the adult microbiota^69^.

### Malnutrition

Food is a major factor shaping the gut microbiota in early life. This is also evident in children who do not receive adequate nutrition during infancy and early childhood. Malnourished infants and children develop an immature gut microbiome^70^, which may contribute to impairment of child growth^71,72^. The disturbance in microbial succession in malnourished children could contribute to lifelong deficits in growth and development as reviewed elsewhere^73^. Intriguingly, a recent 3-month-long intervention study showed that a microbiota-directed complementary food prototype in comparison with an existing ready-to-use supplementary food promoted weight gain in children with moderate acute malnutrition between the ages of 12 months and 18 months of age^74^. The study suggested that the improved weight gain could be mediated by a more complete repair of the gut microbiota^74^. Supporting this hypothesis, a placebo-controlled trial in 2- to 6-month-old undernourished Bangladeshi infants with severe acute malnutrition found that supplementation with a *Bifidobacterium longum* subspecies *infantis* strain promoted weight gain that was associated with reduced levels of intestinal inflammation markers^75^. Globally in 2020, 149 million children under the age of five years were estimated by be stunted^76^, emphasizing the need for also understanding the role of the immature gut microbiome in malnourished children.

## Colonic fermentation and microbial metabolites in early life

**Figure 1.**
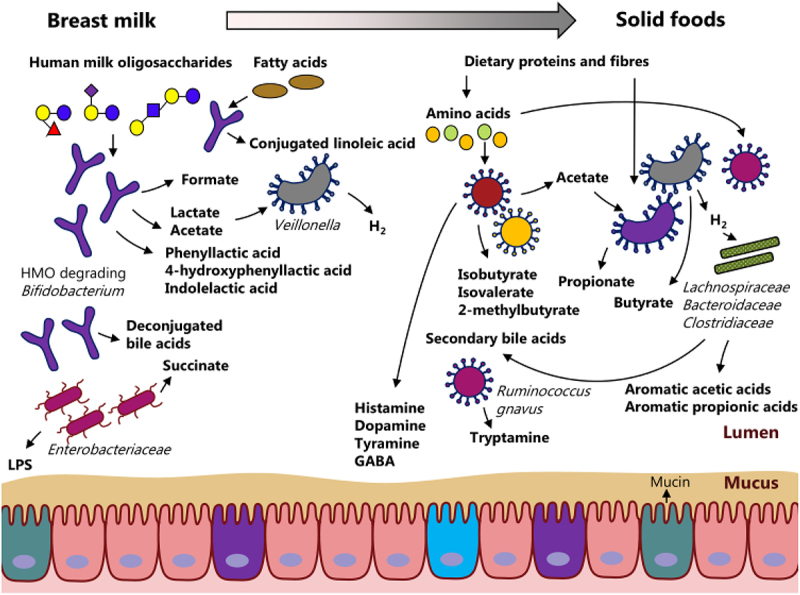
Gut microbiota-derived metabolites in early life. Gut microbial metabolism changes in early life with the progression in early nutrition from breastfeeding to solid foods concurrent with a progression in microbiome and metabolome diversity. During breastfeeding, the dominance of the infant gut by human milk oligosaccharide (HMO) degrading *Bifidobacterium* species results in high levels of lactate and acetate, as well as in aromatic lactic acids (i.e. phenyllactic acid, 4-hydroxyphenyllactic acid and indolelactic acid). With the progression in diet, the dietary complexity increases and more indigestible proteins and fiber end up in the colon of the child. Consequently, colonic fermentation changes resulting in the formation of short-chain fatty acids (SCFA, i.e. acetate, propionate and butyrate) and gases (i.e. hydrogen and methane). Furthermore, proteins are degraded into amino acids, which are fermented by the resident gut microbes into branched SCFAs (i.e. isobutyrate, isovalerate and 2-methylbutyrate), amines (i.e. histamine, dopamine, tyramine, γ-aminobutyric acid (GABA), tryptamine), as well as aromatic acetic and propionic acids (e.g. indoleacetic acid and indolepropionic acid).

The transitioning from a sole source of milk to a diverse range of foods^77^ not only shapes gut microbiome composition^1,8,78^, but also shapes gut microbial metabolism in early life resulting in the generation of a plethora of small molecules (metabolites) (Figure 1).

### Colonic fermentation of carbohydrates

The most studied microbiota-derived metabolites are the SCFAs. SCFAs are products of colonic fermentation of HMOs, dietary fiber and proteins, and known to affect human metabolism in numerous ways via activation of several host receptors in different organs^79^. SCFAs also serve as energy substrates both for the resident gut microbes (as discussed further below), as well as human cells with butyrate being a primary energy substrate for colonocytes^80^, and propionate being a substrate for gluconeogenesis in the intestine^81^ and liver^82,83^. In adults, the major SCFAs acetate, propionate and butyrate are typically found in a 3:1:1 ratio, whereas succinate and lactate appear as intermediates and therefore do not appear in consistent levels. However, in early life, SCFA profiles change concordant with the child’s progression in dietary and microbiome diversity^84,85^. During breastfeeding (early phase), the SCFA profile is characterized by low acetate and high succinate, during complementary feeding (middle phase) by high lactate, pyruvate and formate, and after cessation of breastfeeding (late phase) by high propionate and butyrate^84^. The high abundances of lactate, pyruvate and formate measured in the infant gut during the early phase reflect a less-developed microbiome since these metabolites, in the context of a developed gut microbiome, typically would have been converted into other metabolites^79,86^. Compared to non-breast infants, exclusive breastfed children have been reported to have lower absolute concentrations of total SCFAs including acetate, butyrate, propionate, valerate, isobutyrate, and isovalerate, yet higher concentrations of lactate at four months of age^87^. *Bifidobacterium* are highly abundant in breastfed infants^88^ due to their ability to metabolize HMOs^89^, which results in the production of acetate and lactate^90^. Yet, with the cessation of breastfeeding and development of the gut microbiome^1^, butyrate production increases in the infant gut^84^. Despite the large dynamics in SCFAs observed in early life and their potential importance as signaling molecules, the relationships between SCFAs, the gut microbiome and given carbohydrate substrates are rather limited. Furthermore, with over 200 HMO varieties identified in human milk and with large variations in HMO profiles between mothers and throughout lactation^91,92^, it also remains to be elucidated how differences in breastmilk HMO profiles affect the infant’s gut microbial metabolism.

### Colonic fermentation of proteins

Studies on gut microbial metabolites in the context of early life have mainly been limited to the study of SCFAs^90,93–95^. However, given that intake of dietary fiber and proteins increases with the complementary diet^77,96^, which is associated with increased gut microbial diversity^68^ and capacity to transport amino acids during infancy^8,78^, a multitude of microbial-derived metabolites of dietary fiber and proteins^58,59,97–104^ are likely to change with this dietary transition. For example, it was recently reported that HMO-degrading *Bifidobacterium* species convert aromatic amino acids (i.e. tryptophan, phenylalanine and tyrosine) into aromatic lactic acids (i.e. indolelactic acid, phenyllactic acid and 4-hydroxyphenyllactic acid) in the infant gut^88^. In agreement with breastfed infants being dominated by *Bifidobacterium* ^68^, aromatic lactic acids have been found in higher levels in feces of breastfed infants compared to formula-fed infants^85,105^ and weaned infants^88^, emphasizing the strong interplay between early life nutrition and specific gut microbes affecting the levels of microbial aromatic amino acid catabolites in early life.

Other examples of microbiota-derived metabolites changing in early life are the branched-chain fatty acids (BCFA, i.e. isobutyrate, isovalerate, 2-methylbutyrate). The BCFA reflect increased proteolytic fermentation as they originate from conversion of the branched-chain amino acids valine, leucine and isoleucine^21^, which are implicated in insulin resistance^106^. The levels of proteins and amino acids could likely play a role in dictating the levels of BCFA, since a study found that breastfed infants had significantly lower levels of fecal BCFAs compared to infants fed two infant formulas with extensively hydrolyzed proteins and free amino acids, respectively, at one and two months of age^107^. Infant formula typically contains more protein and lower free amino acids than breast milk^108,109^, suggesting that more proteins are being fermented in the colon of formula-fed infants. In line herewith, a study in newborn rhesus monkeys found that by reducing the protein content in formula, urinary levels of microbial-derived proteolytic compounds (i.e. 3-indoxylsulfate, 4-hydroxyphenylacetate, and 2-hydroxyisobutyrate) could be reduced^110^. Fecal BCFA levels have also been associated with variations in breastmilk lipid and HMO compositions^111^. Furthermore, BCFA have been reported to increase in the infant gut over time independent of early feeding (breastfeeding versus formula feeding)^57,84,93^, suggesting that the lack of HMO following a progression toward solid food promotes a shift toward amino acid fermentation in the infant gut. Also biogenic amines (i.e. cadaverine and tyramine) have been reported in higher levels in feces of formula-fed infants compared to breastfed infants^112,113^. These differences could be due to differences in the gut microbiome composition, as infant formula-associated genera include *Streptococcus* and *Enterococcus*^114^, which are producers of putrescine, tyramine and cadaverine^115,116^. Another prevalent commensal in early life as children progress toward a solid diet is *Ruminococcus gnavus*^117^, which is also able to produce tryptamine *in vitro*^118^. However, it remains to be investigated whether *R. gnavus* indeed produces tryptamine in the infant gut. In fact, many microbial proteolytic-derived metabolites associated with detrimental effects on cardiovascular and metabolic health in adults, such as trimethylamine-N-oxide (TMAO, derivative of carnitine, betaine and choline)^58^, imidazole propionate (derivative of histidine)^59^, phenylacetylglutamine (derivative of phenylalanine)^60^, p-cresol sulfate and indoxyl sulfate (derivatives of tyrosine and tryptophan, respectively)^61^, remain understudied in the context of early life. These metabolites would be expected to arise when children transition toward adult-like solid foods. Yet, very few studies have looked into the impact of the complementary diet on the infant gut microbiome^68^.

### Colonic fermentation of lipids and bile acids

Dietary fat is also metabolized by microbes in the gastrointestinal tract. Infants are, through breast milk and to some extent formula milk, provided with the essential fatty acids linoleic acid (LA) and alpha-linoleic acid (ALA), as well as the long chain polyunsaturated fatty acids (LC-PUFA): eicosapentaenoic acid (EPA), arachidonic acid (AA) and docosahexaenoic acid (DHA, omega-3 fatty acid)^77^. Besides having direct effects on the gut microbiome composition and activity^119^, the polyunsaturated fatty acids can be metabolized by colonic microbes into hydroxy fatty acids, oxo fatty acids, conjugated fatty acids, and partially saturated trans-fatty acids as intermediates^120–122^. Interestingly, specific *Bifidobacterium* strains have been found to be able to convert LA to conjugated linoleic acid (CLA)^122,123^, which has been associated with a variety of systemic health promoting effects^124^. Although the production and importance of CLA in the infant gut are currently unknown, another LA-derivate measured in feces, 12,13-dihydroxy-9Z-octadecenoic acid (12,13-DiHOME) has been suggested to impede immune tolerance in early life^125,126^. This metabolite was recently reported to be produced by common early-life gut microbiome members, namely *B. bifidum* and *E. faecalis* strains^125^. Furthermore, a recent randomized, controlled, clinical trial reported that infants receiving formula milk containing HMOs (i.e. 2-fucosyllactose and LNnT) had reduced fecal levels of two dihydroxy fatty acids (12,13-DiHOME and 9,10-dihydroxy-12Z-octadecenoic acid (9,10-diHOME)) compared to infants receiving control formula milk without HMOs, suggesting that HMO-induced alterations in the microbiome may affect microbial conversion of fatty acids^127^. Therefore, more studies are warranted to investigate intestinal fatty acid metabolism in the infant gut, which is likely to depend on the amount of fatty acids ingested, the efficacy of absorption in the small intestine, as well as the individual gut microbiome composition.

Upon intake of fat, bile is released into the small intestine to aid digestion of lipids. Bile acids constitute the primary component of bile and is conjugated by glycine (dominant in humans) and taurine, respectively^128^. While approximately 95% of the bile acids are reabsorbed in the ileum and returned to the liver via the portal vein, unabsorbed bile acids end up in the colon where they undergo chemical transformations by the microbiota resulting in secondary bile acids^129^. Microbial deconjugation of bile acids reduces their reuptake in the small intestine, which affects the pool of bile acids entering the colon^129^. Furthermore, microbial modification of bile acids affects their chemical structure leading to altered signaling via host receptors including the nuclear farnesoid X receptor (FXR) and G protein coupled bile acid receptor 1 (TGR5)^128^, which may have major impact on host metabolism as discussed elsewhere^130,131^. Concordant with the succession of the gut microbial community in early life, the intestinal bile acid profiles develop according to both the microbiota-stage and nutritional stage. A longitudinal study with 10 healthy Japanese subjects found that during the first six months of life, infants dominated by *Bifidobacterium* species had high fecal levels of unconjugated primary bile acids whereas infants dominated by *Enterobacteriaceae* had high fecal levels of conjugated primary bile acids^132^. Indeed, bile salt hydrolase activity, catalyzing deconjugation of conjugated bile acids, is common in *Bifidobacterium*^133^, suggesting that *Bifidobacterium* is a key player deconjugating bile acids during breastfeeding. Yet, as infants progress toward a solid diet, dietary energy intake goes up^77^, dietary complexity and gut microbial diversity increase^68^. Consequently, both the total excretion of bile acids in the feces as well as the fecal levels of secondary bile acids increase^85,132^. Furthermore, decreasing level of primary bile acids and an increase of secondary bile acids in feces have consistently been observed after weaning^132,134,135^. Differences in fecal bile acid profiles have also been observed when comparing breastfed and formula-fed infants with breastfed infants showing higher levels of sulfated bile acids and formula fed infants showing higher levels of secondary bile acids^85^. Finally, a study in mice has shown that oral administration of bile acids to newborn mice accelerate postnatal microbiome maturation^136^, suggesting that the progression in intestinal bile acid profiles during infancy also drives maturation of the postnatal gut microbiome.

## Microbial metabolites as modulators of the microbiome community structuring

**Figure 2.**
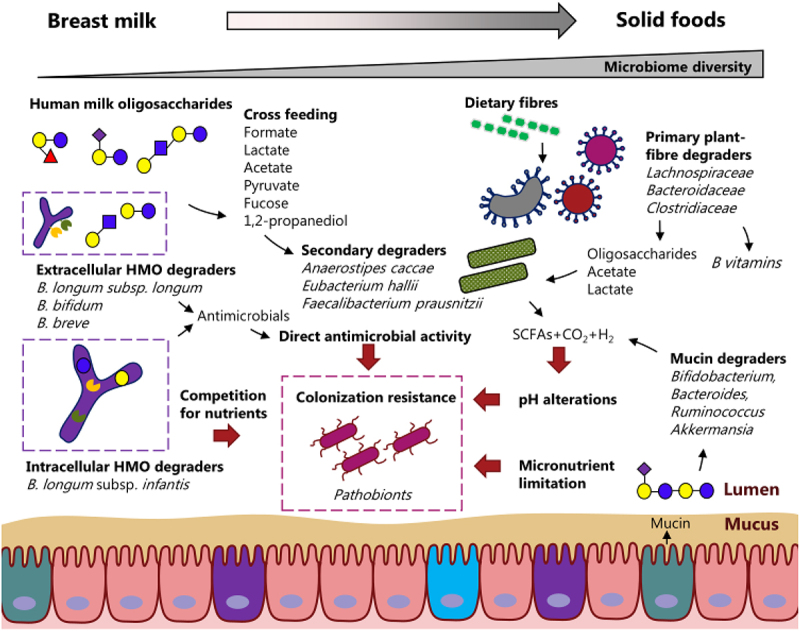
Microbial metabolites as modulators of the infant gut microbial ecosystem. An illustration of the ways in which microbial metabolites are thought to impact the development of the gut microbiome ecosystem during infancy. Human milk oligosaccharides (HMOs) of breast milk are likely to enter the distal gut and be degraded by HMO degraders (for example *Bifidobacterium Bacteroides*, *Ruminococcus* and *Akkermansia*). Among the HMO degraders, particular *Bifidobacterium* species are important as they are specialized in HMO degradation. Some *Bifidobacterium* species (mainly *B. longum* subsp. *longum*, *B. bifidum* and *B. breve*) degrade HMOs extracellular, whereas other *Bifidobacterium* species (mainly *B. longum* subsp. *infantis*) take up the HMOs via ABC transporters and metabolize them inside the cell. The sharing or lack of sharing of nutrients (both carbon sources and micronutrients) have consequences for cross-feeding and competition in the infant gut microbiome affecting the microbial community structure. As the child transitions from a milk-based diet to a solid food-based diet, the microbiome diversity increases and the cross-feeding networks change. Other primary degraders of dietary fibers (typically belonging to *Lachnospiracea, Bacteroideacea, Clostridiacea*) take over and replace the *Bifidobacterium* species. Generation of specific microbial metabolites may be key for colonization resistance toward pathobionts when microbiome diversity is low. The pathobionts may be excluded or kept low in abundance through low intestinal pH (due to short-chain fatty acids, SCFAs), through direct antimicrobial activity such as bacteriocins, which are antimicrobial, or simply through nutrient limitations.

Gut microbial conversion of dietary substrates into metabolites represents a potential regulatory mechanism by which specific gut microbes impact the wider gut ecosystem (Figure 2). In particular HMO-derived metabolites produced by primary HMO degraders drive cross-feeding networks that facilities growth of other early life taxa and crucial interspecies interactions^137,138^. HMOs are poorly digested by the infant but are favored growth substrates for certain *Bifidobacterium* species and strains that can enzymatically degrade these complex dietary components that include LN(n)T, fucosylated and sialylated structures^139^. Along with *Bifidobacterium*, *Bacteroides*, *Ruminococcus* and more recently *Akkermansia* have also been shown to degrade these complex carbohydrates, although in most cases this relates to enzymatic cleavage of sugar side chains and correlates with associated mucin degradation pathways^140–142^. Certain *Bifidobacterium* species seem to have limited impact on wider ecosystem structuring due to their ‘selfish’ digestion of HMOs. This is particularly true for *B. longum* subsp. *infantis* strains where they actively transport (via ABC transporters) and internalize whole (intact) HMOs to perform intracellular breakdown, utilizing the monosaccharide metabolites for their own cellular processes, rather than sharing among other early life microbes^143^. Other species such as *B. bifidum*, *Bifidobacterium pseudocatenulatum*, *B. longum* subsp. *longum* and *B. breve* also carry out extracellular hydrolysis of complex HMOs, and then import and metabolize the resulting mono- and di-saccharides. These strains therefore provide an important keystone function during early life by providing simpler metabolites for cross-feeding^144,145^. Metabolites that can be used by other secondary degraders include the SCFAs acetate and lactate, formate, succinate, pyruvate, fucose, and 1,2-propanediol (1,2-PD)^84,146^. Previous work has indicated that within ‘mini’ *Bifidobacterium* ecosystems (from individual infants), there is often a keystone member that actively breakdown different HMOs, which provides key metabolites such as fucose, galactose, acetate and N-acetylglucosamine then utilized by other non-HMO degrading strains^138^. Other work supports this concept, showing that during growth on HMOs, cross-feeding interactions between multiple bifidobacterial strains increase overall cell numbers, which was linked to production of formate, acetate, 1,2-propanediol, and lactate during co-cultivation^147^. Most recently, a study has indicated how ‘timing’ of sugars in the infant gut shape the wider *Bifidobacterium* ecosystem, with *B. bifidum* and *B. longum* subsp. *infantis* dominating through inhibitory priority effects based on their ability to digest HMOs, whilst *B. breve* benefits from facilitative priority effects and dominates by utilizing fucose (aligning with profiles observed in metagenomic datasets)^148^. These studies highlight the importance of cross-feeding term ‘syntrophy’ for *Bifidobacterium*, which has been defined as ‘obligately mutualistic metabolism’ whereby without a complete mini ecosystem all or combinations of strains cannot survive without the other. This collective approach to HMO utilization may indicate additional processes by which multiple *Bifidobacterium* species and strains dominate the breastfed infant gut, and successfully outcompete other bacterial taxa. Indeed, acetate and lactate production by *Bifidobacterium* (in response to HMO-metabolism) is associated with lowering intestinal pH, which also correlates with changes in microbial composition, such as reducing levels of potential pathogens like *Enterobacteriaceae*, thus enhancing colonization resistance in the early life gut^149,150^. In agreement, *in vitro* experiments have found that SCFAs directly inhibit growth of enteric pathogens belonging to the *Enterobacteriaceae* family including *Escherichia coli, Klebsiella and Salmonella* in a pH-dependent manner^151–153^.

Looking beyond *Bifidobacterium*, HMO-derived metabolites also cross-feed other early life taxa. *In vitro* studies have shown that growth of *Limosilactobacillus reuteri* is enhanced through 1,2-propanediol produced by *B. breve* UCC2003^146^. There has also been a number of studies exploring the cross-feeding impacts on a range of butyrate-producing bacteria, given their key health-promoting role. Specifically, butyrate-producing bacteria can utilize acetate and lactate, and HMO-derived monosaccharides as carbon sources, which serves to increase their levels in the infant gut. For example, *Anaerostipes caccae* has previously shown to only grow in co-culture with *B. longum* subsp. *infantis*, leading butyrate production^154^. Additional SCFAs production by *Eubacterium hallii* in response to (fucosylated) HMO breakdown by *B. breve* and/or B. *longum* subsp. *infantis* enables utilization of L-fucose (for production of butyrate), and 1,2-PD uptake for propionate production^137^, although the latter has yet to be demonstrated in the infant gut. The availability of L-fucose also supports growth of other microbes encoding Fuc catabolic pathways such as *B. thetaiotaomicron* and *A. muciniphila*, but these microbes can also cleave fucose from fucosylated HMOs, according to their fucosidase activity^155^. Notably, some pathogenic bacteria have evolved strategies to use fucose to their advantage, including *Salmonella* and *C. jejuni*, which is the latter case appears to be enriched in breastfed infants when compared to those on formula^156^. Thus, the availability of different metabolites in the gut may both have beneficial and detrimental consequences depending on the ecological circumstances.

In some cases there may be competitive interactions via production of metabolites know to inhibit metabolic reactions and growth of other microbiota members e.g. hydrogen sulfide (H_2_S), or SCFAs (lowering the pH), or via production of metabolites with direct antimicrobial activity such as bacteriocins. Bacteriocins are ribosomally-synthesized antimicrobial peptides, which are active against other bacteria, and can be either narrow spectrum (i.e. targeting similar species) or broad spectrum (i.e. across diverse genera), with the host bacterium being resistant^157^. A number of early life microbiota members, including *Bifidobacterium*, are known to produce these antimicrobials which may be linked to their ability to modulate the wider microbiota, and also inhibit overgrowth of potentially pathogenic bacteria that would otherwise be harmful to the infant^158^. The gene clusters required for biosynthesis appear to be very strain specific; previous work has indicated that the bacteriocin bifidin (produced by *Bifidobacterium bifidum* NCDC 1452) has activity against a range of Gram positive and Gram negative’s including *Escherichia coli* and *Staphylococcus aureus*^158^, which are common members of the early life gut microbiota. There are other *Bifidobacterium*-associated bacteriocins, however these have shown activity against specific food-borne pathogens including *Salmonella* spp. and *Bacillus cereus*, thus may not be so relevant in the context of the first 12 months of life. More recently, *Bifidobacterium longum* subsp. *infantis* LH_664 has been shown to encode and produce a narrow spectrum bacteriocin (putatively named Bifidococcin_664) that whilst not impacting growth of *Lactobacillus* and *Enterococcus* strains, does have discrete activity against a potentially important pathobiont in the (preterm) gut, *Clostridium perfringens*^159^. The ability of these active peptides to target specific bacteria, may be particularly important in the context of early life perturbations (such as preterm birth, C-section and/or antibiotic treatments), when levels of pathobionts may be higher given the absence of these key metabolite/peptide modulating factors that would otherwise be produced by *Bifidobacterium* which may be depleted in these contexts. However at this stage it is unclear if the dietary (e.g. HMO) milieu in the gut plays a role in shaping the production of these peptides, and further work is required to disentangle these relationships.

With the introduction of additional complex dietary components (particularly plant-derived) during weaning there is a significant expansion in cross-feeding networks due to the resultant complex milieu of metabolites produced by primary degraders^144,145^. This is due to the huge variety of glycoside hydrolases encoded across different taxa (particularly in the *Bacteroides* genus, many of which act as primary degraders) that enables the diverse array of bonds between monosaccharide units to be ‘broken’, thus allowing these simple sugars to enter into different metabolism pathways to provide energy (ATP) for cellular responses and growth^160^. Fermentation end products are further metabolized by cross-feeders which often results in the production of SCFAs, with increasing production of butyrate and propionate, in comparison to the acetate dominant SCFA profile observed during exclusive milk-based feeding^161^. Additional key components that can be produced as a result of protein fermentation are amino acids, which play a central metabolism (enzyme co-factors) role for many bacteria, driving growth and changes in motility, biofilm formation and virulence factor expression^162^. Furthermore, there are strain level differences with respect to B vitamins biosynthesis, which is required by the host, and by other strains unable to *de novo* synthesize these key metabolites^163^. For example, several early life taxa are predicted, and have been experimentally confirmed, to produce vitamin B1 including some *Bacteroides* spp., *Clostridium* spp., *Lactobacillus* spp., and *Bifidobacterium* spp.^164^. These strains are expected to play a key role for other species and strains that lack a vitamin B1 synthesis (e.g. *Faecalibacterium* spp.), highlighting the cross-feeding and also competition for this essential metabolite in the infant gut^165^. It is clear that this time-period represents a highly dynamic period of ecosystem development, with burgeoning cross-feeding networks being established, and like other underdeveloped ecosystems, may be particularly sensitive to diet-derived perturbations^166^. However, clear evidence on the longer-term effects during this time period are required via both longitudinal cohorts and experimental studies. For a comprehensive review of this with a more complex (adult-like) ecosystem see a review by Oliphant and Allen-Verco^161^.

Finally, the spatial context of these potential metabolite interactions should be considered. Clearly the levels and diffusability of key metabolites would be expected to have the most pronounced impact on ecosystem structure on those microbes in close proximity to each other. However, given the complex ‘geography’ of the gut, including the overall length, mucus layers, and micro-structure e.g crypts, it is likely that the studies undertaken so far may not completely replicate these spatial considerations. Therefore, additional model systems e.g. organoids and mice, may be required to pinpoint relative contributions of microbial metabolites as modulators of the infant gut microbiome.

## Microbial metabolites as mediators of host-microbial cross talk in early life

**Figure 3.**
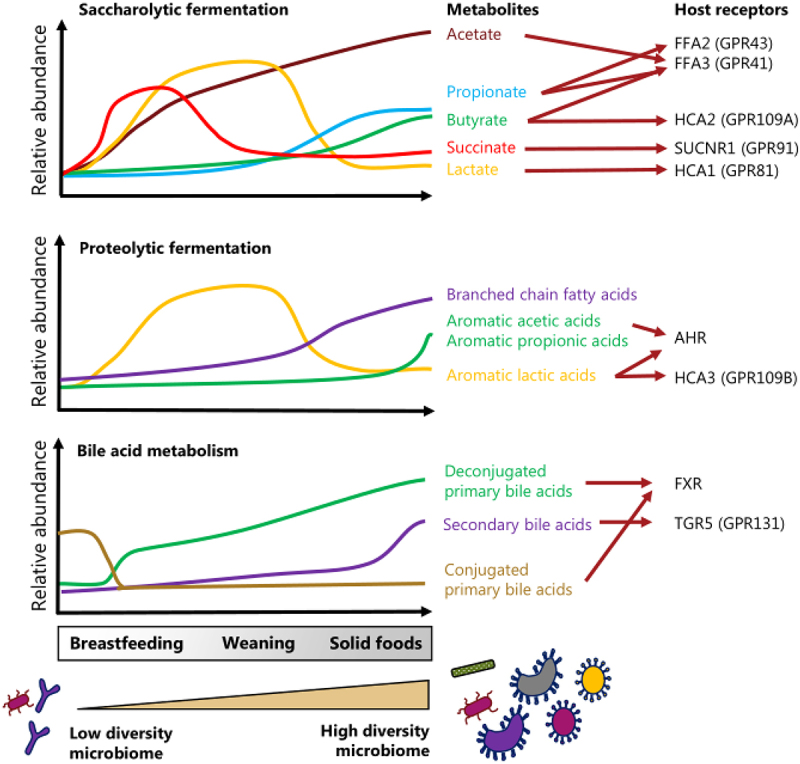
Dynamics of selected signaling metabolites in the infant gut and their interaction with host receptors. The figure illustrates the change in relative abundances of known key microbiota-derived metabolites generated from saccharolytic^84^ and proteolytic fermentation^84,88^ as well as from bile acid metabolism^132^ in the infant gut. The metabolite profiles change as the infant goes from breastfeeding to solid foods concurrent with a maturation of the gut microbiome from a less to a more diverse microbiome. The main host receptor targets for each of the signaling metabolites is indicated to the right. As indicated by the arrows, some metabolites can bind several different host receptors^168^.

**Figure 4.**
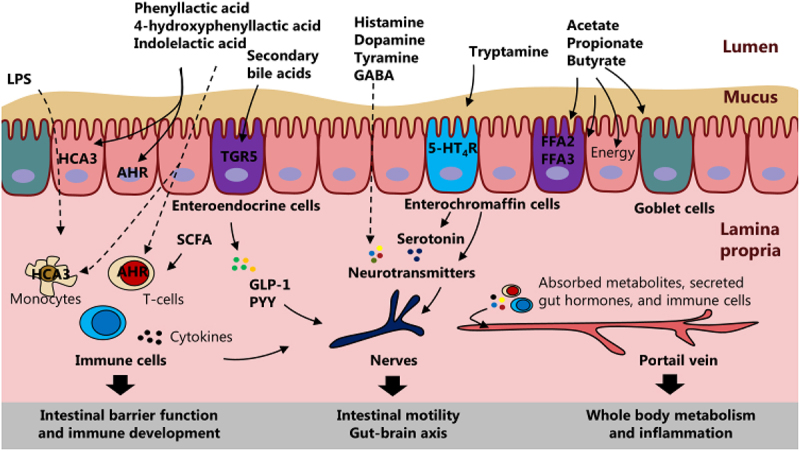
Gut microbiota-derived metabolites as mediators of host-microbial cross-talk in early life. The microbiota-derived metabolites can impact host development in various ways as they activate different host receptors. For example, microbiota-derived metabolites can stimulate the intestinal immune system (affecting release of cytokines), enteroendocrine cells (affecting release of gut hormones), and enterochromaffin cells (affecting release of serotonin). Subsequently, the secreted molecules can affect the intestinal barrier and immune function, the intestinal nervous system with effects on intestinal motility and the gut-brain axis, and potentially the host metabolism and inflammation throughout the body upon absorption and circulation through the portal vein.

Given the dynamics of microbial metabolites in the gut and their absorption into the circulation^167^, these microbiota-derived molecules not only shape the gut microbiome ecosystem, but also likely play a central role in mediating host-microbial cross-talk in early life as they may communicate with host cells via host receptors in the intestine and distant organs (Figure 3)^21,168,169^. However, only few studies have studied the gut luminal and circulating concentrations of the metabolites in early life. This is an important gap to fill in order to figure out whether concentrations of the metabolites are within the range of the dose-response curve for the particular metabolite on the host receptor. Here we shortly discuss four areas where microbial metabolites may play a vital role in early life (Figure 4).

### Microbial metabolites and intestinal barrier function

Although a ‘strong’ barrier is considered essential for host health, during very early life a balance is required; allowing key microbial metabolites to cross and initiate immune programming in the underlying mucosal and at more distal and systemic sites. Butyrate is the main ‘fuel’ used by intestinal epithelial cells (IECs) for growth^170^, however concentrations of butyrate are relatively low during milk feeding, but steadily increase across the weaning period and beyond^84^, due to the expansion in butyrate producing bacteria given the increasingly complex dietary environment^84^. Acetate (and propionate) can be consumed by IECs, however this is a less efficient process than for butyrate. Nevertheless, acetate is also associated with inducing additional anti-inflammatory responses in the gut epithelium such as enhancing tight junction protein expression and anti-inflammatory cytokine production^171^. This appears to be mediated through Toll-like receptors (TLRs) and G protein-coupled receptors (GCPRs), including FFA2 pathway stimulation, as defined using KO mouse models. This acetate-ligand interaction can lead to NLRP3 inflammasome activation^172^, which also links to a more efficient clearance of enteric pathogens from the gut^173^, and enhanced production of sIgA (via Dendritic Cell-B cell activation). Of the other metabolites discussed above, such as B vitamins, it is appreciated these also directly modulate host immune responses, including the epithelial barrier (for a comprehensive overview see Yoshii *et* *al*.^174^, and there is also work suggesting that bacteriocins may have a dual role, providing direct antimicrobial activity and also promoting immune responses, however this seems to be more with respect to innate immune cells like macrophages^159^. Furthermore, aromatic lactic acids could be important in early life since they may modulate immune function via both the aryl hydrocarbon receptor (AhR) and hydroxycarboxylic acid receptor 3 (HCA3)^88,175,176^. During the last decade, tryptophan catabolites have emerged as potential key AhR-ligands playing a role in regulating intestinal barrier and immune function^98^. It was recently shown that fecal levels of indolelactic acid were positively correlated with the fecal samples’ ability to activate AhR in an AhR-reporter cell line^88^, suggesting that indolelactic acid is a key AhR-ligand in early life. However, it remains to be determined whether indolelactic acid and/or other microbiota-derived AhR ligands indeed play a role for immune barrier development in the context of early life. For all of these studies, it is important to note that most work in this area has concentrated on adult models. Previous work has suggested that microbes (including *Bifidobacterium*) drive distinct responses in the neonate vs. later life time points^177^, thus there is a pressing need to determine how microbial metabolites such as aromatic lactic acids and SCFAs modulate host intestinal responses during the early life developmental window.

### Microbial metabolites and intestinal motility

Stool frequency and consistency vary significantly according to early life nutrition between and within infants during the first year of life^178^. Despite this, very little is known about the interplay between the diet, the developing gut microbiota, and the ever-changing bowel habits in early life. Several microbial metabolites, which have been measured in the infant gut, could potentially play a role in affecting gut motility in early life, although this has not yet been investigated. For example, tryptamine has been found to accelerate transit time *in vivo* through the 5-HT4 receptor (5-HT4R), a receptor uniquely expressed in the colonic epithelium^118,179^. Other tryptophan-derived catabolites may also affect intestinal motility via AhR signaling in enteric neurons in the distal gastrointestinal tract^180^. Furthermore, SCFAs can stimulate the release of serotonin (5-HT) in enterochromaffin cells by activating GPCRs such as FFA3 (GPR41), FFA2 (GPR43), and HCA2 (GPR109A). Serotonin has multiple paracrine and endocrine roles including promotion of peristalsis via the enteric nervous system^181^. Secondary bile acids and SCFAs may also through TGR5 and FFA2/FFA3, respectively, stimulate release of GLP-1 and PYY in enteroendocrine cells and thereby affect colonic motility^182,183^. Variations in intestinal transit time have been associated with the gut microbiome composition and activity in adults^184^. Furthermore, given the large fluctuations and changes in SCFAs, secondary bile acids and proteolytic metabolites during the first year of life, it is possible that the gut microbiome contributes to inter- and intra-individual variations in bowel habits during infancy. Such variations could potentially be linked to common functional gastrointestinal disorders in infants such as infantile colic and functional constipation^185^.

### Microbial metabolites and whole body metabolism

SCFAs are proposed to affect whole body metabolism and inflammation upon absorption and circulation in the blood stream to distant organs through interactions with host receptors^169^. Furthermore, SCFAs can indirectly affect whole body metabolism by stimulating secretion of gut hormones (e.g. GLP-1 and PYY) in enteroendocrine cells affecting appetite and through stimulation of immune cells that regulate whole body metabolism^79^. The high gut luminal levels of SCFAs, lactate, and succinate measured during infancy could possibly be important for recruitment of macrophages and neutrophils acting through FFA2, HCA1, and GPR91, respectively^168^. However, in the context of early life, little is known about the role of SCFAs regulating whole body metabolism and inflammation. High fecal levels of formate during early infancy (3–4 months of age) have been associated with lower BMI z-score, whereas high fecal levels of butyrate have been associated with a higher BMI z-score at 3 years of age in Canadian infants^186^. Based on these observations one could speculate that the development of the infant gut metabolome from high formate (reflecting a low-diversity microbiome) to high butyrate (reflecting a more diverse microbiome) could play a role in growth trajectories^186^. Yet, the timing of the gut metabolome development may be important, since another study found that Danish infants with normal weight gain at nine months of age had significantly higher fecal concentrations of butyrate compared to infants with high weight gain^111^. In line with this, both exclusive formula feeding as well as early introduction to solid foods (vs later) in infancy has been associated with altered gut microbiota and increased risk of being overweight in early childhood^52,187^. Prospective studies focusing on the infant gut metabolome development are warranted to understand whether the gut microbiota via production of metabolites is indeed casually involved in the programming of host metabolism, which may trigger long-lasting physiological effects that promote or prevent diseases.

## Concluding remarks

Together, there is a clear need to uncover and explore microbial metabolites and their interactions with both gut microbes and host cells within early life. These microbial interactions may be pivotal for establishment of the gut microbiome and appropriate cross-talk between gut commensals and host cells with both short- and long-term physiological consequences. Currently, much of the work with regards to microbial metabolites and ecosystem structuring have been done in the context of the adult gut. Furthermore, only few aspects of cross-feeding in the infant gut such as *Bifidobacterium-*metabolism of HMOs and the generation of SCFAs have been studied. Therefore, prospective infant cohort studies following infants’ progression in diet, concurrent with their progression in gut microbiome composition and activity, are needed to further advance the field. By collecting and analyzing longitudinal fecal samples by untargeted metabolomics, unpreceded insights into known and novel microbial-derived metabolites, as well as their dynamics across and within infants during early life, can be provided^88^. Combined with high-resolution microbiome profiling, enabling strain level classification^20^, as well as immune cell profiling^176^, this may take the field to a whole new level. Furthermore, there is a need for relevant animal models and human organoids mimicking the context of early life to uncover the functional and mechanistic impact of metabolites on host development and specific pathways. Such insights are needed to develop/establish targeted strategies that contribute to healthy growth and development in infants, which may include manipulation of the infant gut microbiota using prebiotics, probiotics or postbiotics. However, before we can tackle these more translational aspects, we need to gain deeper insights into the range and mode-of-action of key microbial metabolites in the infant gut.

## Data Availability

Data sharing is not applicable to this article as no new data were created or analyzed in this study.
